# Diversifying selection of the anthocyanin biosynthetic downstream gene *UFGT* accelerates floral diversity of island *Scutellaria* species

**DOI:** 10.1186/s12862-016-0759-0

**Published:** 2016-09-17

**Authors:** Bing-Hong Huang, Yi-Wen Chen, Chia-Lung Huang, Jian Gao, Pei-Chun Liao

**Affiliations:** 1 Department of Life Science, National Taiwan Normal University, No. 88, Ting-Chow Rd, Sec 4, Taipei, Taiwan 11677 Republic of China; 2 Department of Biological Science and Technology, National Pingtung University of Science and Technology, No. 1, Shuefu Road, Pingtung, Taiwan 91201 Republic of China; 3 The Key Laboratory for Silviculture and Conservation of Ministry of Education, College of Forestry, Beijing Forestry University, No.35, Tsinghua East Road, Beijing, 100083 China

**Keywords:** Anthocyanin biosynthetic pathway, *CHS*, Floral diversity, Island species, Positive selection, *UFGT*

## Abstract

**Background:**

Adaptive divergence, which usually explains rapid diversification within island species, might involve the positive selection of genes. Anthocyanin biosynthetic pathway (ABP) genes are important for floral diversity, and are related to stress resistance and pollination, which could be responsible for species diversification. Previous studies have shown that upstream genes of ABP are subject to selective constraints and have a slow evolutionary rate, while the constraints on downstream genes are lower.

**Results:**

In this study, we confirmed these earlier observations of heterogeneous evolutionary rate in upstream gene *CHS* and the downstream gene *UFGT*, both of which were expressed in *Scutellaria* sp. inflorescence buds. We found a higher evolutionary rate and positive selection for *UFGT*. The codons under positive selection corresponded to the diversified regions, and the presence or absence of an α-helix might produce conformation changes in the Rossmann-like fold structure. The significantly high evolutionary rates for *UFGT* genes in Taiwanese lineages suggested rapid accumulation of amino acid mutations in island species. The results showed positive selection in closely related species and explained the high diversification of floral patterns in these recently diverged species. In contrast, non-synonymous mutation rate decreases in long-term divergent species could reduce mutational load and prevent the accumulation of deleterious mutations.

**Conclusions:**

Together with the positive selection of transcription factors for ABP genes described in a previous study, these results confirmed that positive selection takes place at a translational level and suggested that the high divergence of ABP genes is related to the floral diversity in island *Scutellaria* species.

**Electronic supplementary material:**

The online version of this article (doi:10.1186/s12862-016-0759-0) contains supplementary material, which is available to authorized users.

## Background

Floral color patterns are recognized ecologically important factors that influence reproductive isolation and the response to environmental variation [1]. Anthocyanins form a group of plant pigments that contribute to plant diversity in colorful flowers, leaves, stems, etc. Their production typically starts with chalcone synthesis by naringenin-chalcone synthase (CHS, EC 2.3.1.74), followed by a serial synthesis of flavanones, 3-OH-flavanones, flavan-3,4-diols, and anthocyanidins by the core-structural enzymes chalcone isomerase (CHI, EC 5.5.1.6), flavonone 3-hydroxylase (F3H, EC 1.14.11.9), dihydrokaempferol 4-reductase (DFR, EC 1.1.1.219), leucocyanidin oxygenase (LDOX, EC 1.14.11.19, synonym: anthocyanidin synthase, ANS), and UDP-glucose:flavonol 3-O-D-glucosyltransferase (UFGT, EC 2.4.1.91) (cf. [2]). Upstream and downstream genes of the same pathway might have different evolutionary scenarios. For example, the *UFGT* genes, which are downstream genes in the ABP (anthocyanin biosynthetic pathway) genes, have relatively higher non-synonymous replacement rates than the upstream genes [2]. Different evolutionary mechanisms shape the genetic diversity of the genes that are upstream and downstream of ABP [3]. Selective constraints and codon usage bias are prominent in *CHS*-*D*, while relaxed constraints and more insertion/deletion (indel) events have led to higher *UFGT* genetic diversity in *Ipomoea* sp. [3]. Several studies have shown that upstream *CHS* has a low non-synonymous substitution rate (dN) and this suggests that it evolves with stronger selective constraints than downstream genes, which have an elevated dN [3, 4]. The anthocyanin-regulating transcription factors (TFs) have both a high dN and a synonymous substitution rate (dS), which suggests that there has been a relaxation of selective constraints, but not of positive selection due to ω (=dN / dS) < 1 [5–7]. It has also been suggested that floral diversification is mostly caused by downstream gene diversity [8, 9] and/or diversity in the corresponding TFs [10–12], and that regulatory gene variation is more important than structural mutation for floral anthocyanin diversity in rapidly evolving species [13]. However, mutations of upstream genes (e.g., *CHS*) typically cause large physio-ecological changes, e.g., the elimination of nearly all flavonoids by the suppression of *CHS* [14, 15]. They have also been shown to affect pollination [15, 16].

Adaptation could proceed through changes in either TF or structural enzymatic genes, or a combination of the two types [17]. Our previous study showed that the TF R2R3 type MYB11, which regulates the ABP genes *PHENYLALANINE AMMONIA LYASE* (*PAL*), *CINNAMATE 4-HYDROXYLASE* (*C4H*), *CHS*, *CHI*, and *UFGT* [18], is expressed in the inflorescence buds and is positively selected for in recently diversified Taiwanese *Scutellaria* species (Lamiaceae) [10]. The signatures for positive selection pressures on anthocyanin-regulating TFs indicated that the diversification of Taiwanese *Scutellaria* species is affected by transcription-level evolutionary selection, but it is still unknown whether structural enzyme-level selective pressure (e.g., relaxation of selective constraints or positive selection) is involved. Taiwanese *Scutellaria* species have arisen many times in mainland China, particularly around *c.* 0.610 Mya, and have had a short species divergence time. This was followed by very recent within-island speciation events that occurred between 0.204 Mya and 0.015 Mya [19]. Taiwan is a “semi-isolated” island that is separated from continental Asia by the Taiwan Strait, where the narrowest distance between China and Taiwan is 130 km. It was repeatedly connected to continental Asia during glaciation periods, but became isolated during interglacial periods. Repeated connection with the continent enriches species diversity [20]. In addition, steep and rugged terrains create diversified environments and increases opportunities for adaptive radiation.

The phylogenetically close Taiwanese *Scutellaria* species are distinguished by different corolla colors and spots and their differential growth habitats [21]. The varied floral color pigmentations are often determined by ABP genes and their regulators [11, 22]. These rapidly evolving species may show differential expression and cryptic genetic variation in response to different environmental conditions [23]. The presence of cryptic genetic variation suggests that the plants exhibit low phenotypic variance under typical environmental conditions, but diverge in their response (i.e., high phenotypic divergence) to small genetic variations. For example, differentiation copigment composition and ABP regulator under stress may alter the accumulation or colour pattern of anthocyanins isolated from *S. baicalensis* [18, 24, 25]. Besides, norms of reactions from different genotype of ABP genes are often associated with environmental adaptation, e.g., light damage [26], autumnal senescence [27], and pollination syndrome [28]. Moreover, arising new adaptive genotype with different norm of reactions may lead adaptive radiation [29]. These adaptive genotypes could also be geographic structured due to natural selection and leaded to flower color diversity [30] and may cause variation of evolutionary rate in ABP gene in different geographic context. Therefore, we can expect that the adaptively evolving genes (e.g., ABP genes in Taiwanese *Scutellaria* species) will exhibit high ω (dN > dS) in rapidly evolving species, but not in other species. In contrast, if ω is similar between species, then they have similar patterns of molecular divergence.

In this study, we investigated whether the rapid divergence in the floral patterns of *Scutellaria* species is similar to the case study of *Ipomoea* [3] that the flora diversity is a result of the relaxation of selective constraints on genes that are downstream of ABP. We also asked a question that if the rapid divergence of Taiwanese *Scutellaria* species is associated with adaptive evolution, whether the signatures of positive selection could be detected on ABP genes. By assessing the genetic diversity of these island species, we offer a case of positive selection that accelerates the rate of gene evolution, which may explain the morphological diversification in a group of recently divergent species.

## Methods

### Sampling

To test the neutrality of ABP genes in Taiwanese *Scutellaria*, all eight of the native Taiwanese *Scutellaria* species and an additional 23 species were sampled either from the field or from a seed bank such as B & T World Seed (Additional file 1: Table S1). All plants were grown in a greenhouse at the National Taiwan Normal University, Taipei, Taiwan, and the leaves were collected for DNA extraction.

### Molecular techniques

To obtain the *CHS* and *UFGT* sequences, a polymerase chain reaction (PCR) analysis was performed in a MultiGene thermal cycler (Labnet International, Inc., Woodbridge, NJ, US) using 20 ng template DNA, 0.5–1 U Taq (Bernardo Scientific Corp., Taipei, Taiwan), 100 μM deoxyribonucleotide triphosphate, and 0.2 μM of each primer. Primers for *CHS* amplification were adapted from those used by Chiang et al. [19]. The primers for *UFGT* amplification were designed from the transcriptomic assembly of inflorescences buds used by Huang et al. [15]. The primer sequences for *UFGT* were ScUGT-F1: 5′-GTTCCAATGATAGCTCATGG-3′ and ScUGT-R1: 5′-GGAACATAGGCACTCAATTC-3′. The PCR program was set to 94 °C for 3 min for enzyme activation, followed by 35 cycles at 94 °C for 40 s, melting temperature for 40 s, and 72 °C for 80 s, with a 5-min final extension at 72 °C. The PCR products were sequenced directly in both directions using an ABI BigDye 3.1 Terminator Cycle Sequencing Kit (Applied Biosystems, Foster City, CA, USA). All sequences were visually checked via chromatograms using an ABI PRISM®3730XL DNA Sequencer (Perkin-Elmer, Foster City, CA, USA).

### Phylogenetic analyses

Sequence alignments were conducted using the MUSCLE multiple sequence alignment software tool [31, 32]. Phylogenetic analyses were performed using the neighbor joining, maximum likelihood, and Bayesian method with the assistance of MEGA v.6 [33], PhyML v.3.0 [34], MrBayes v.3.2 [35]. The best nucleotide substitution models for *CHS* and *UFGT* were the Tamura 3-parameter, with an assumption of a certain fraction of evolutionary invariable sites (T92 + G, gamma shape parameter 0.23), and the Kimura 2-parameter with a discrete gamma distribution (K2P + G, gamma shape parameter 0.42), respectively. Besides, T92 + G model was not available in maximum likelihood and Bayesian methods, so the second best model Hasegawa-Kishino-Yano with a discrete gamma distribution (HKY + G, gamma shape parameter 0.23) was adopted. These were determined by the Bayesian Information Criterion (BIC) method. Pairwise deletions for the treatment of gaps and 1000-times bootstrap replication were set for the phylogenetic reconstruction. To track the corolla colour evolution in skullcaps, we applied methods that originally designed for biogeographic history with consideration of ‘vicariant speciation’ to infer the trait evolution. Three methods were used: the dispersal-vicariance analysis (DIVA), dispersal-extinction-cladogensis analysis (DEC), and BAYAREA method incorporating with founder-event parameter *j* [36]. In these analyses, ecological characters are analogues to the ancestral distributions and ecological events are analogous to speciation events [37]. These analyses were conducted in BioGeoBEARS [38].

### Detection signals for positive selection

To understand the degrees of genetic divergence within island-based evolving species (implying different selective pressure), the equality of means of evolutionary divergence over Taiwanese species (T/T) pairs dN/dS (ω) for *CHS* and *UFGT* was compared to the ω between non-Taiwanese and Taiwanese species (nT/T), and the non-Taiwanese species (nT/nT) Codon-based maximum likelihood models of ω were constructed by the codeml program in PAML v.4.2 [39] and the HyPhy package [40]. First, we estimated the averaged ω from the PAML Model M0, which describes a single ω for all sites. Distributions of pairwise ω values for *CHS* and *UFGT*, T/T, nT/T, and nT/nT were compared using Student’s *t*-test. We also drew a dN-vs.-dS plot and examined the rate saturation of non-synonymous and synonymous mutations. An ω-vs.-dS plot was constructed to illustrate the relative time series of selective pressures, where dS was the time index and ω was the indicator of selection pressure [41]. Besides, ω values were also obtained from ratio of nonsynonymous and synonymous substitution rate calculated from relative rate test implemented in HyPhy packages [40]. Sequences from *Tinnea rhodesiana* (Lamiaceae) were used as outgroup in relative rate test and ω values were compared using paired *t*-test. Finally, AMOVA analysis implemented in Arlequin [42] was conducted to confirm the geographic structure of both *CHS* and *UFGT* in Taiwanese and non-Taiwaneses species. The sliding-window analysis of the ω values between Taiwanese and non-Taiwanese species was performed using DnaSP v.5 [43], and the secondary structures were predicted using the Garnier-Osguthorpe-Robson method [44, 45]. The sliding window analyses and secondary structure predictions help improve understanding of the divergent regions and their corresponding secondary structure. To further confirm signature of positive selection in *CHS* and *UFGT* in Taiwanese sister species, McDonald and Kreitman (MK) test [46] were conducted. We further checked whether the polymorphisms of *CHS* and *UFGT* of Taiwanese species attribute to ancestral polymorphism, i.e., the balancing selection, polymorphisms of *CHS* and *UFGT* within and between Taiwanese and non-Taiwanese species were compared using Hudson, Kreitman and Aguade (HKA) test [47]. Both MK and HKA tests were implemented in DnaSP v.5 [43].

The branch model (free-ratio) was tested against the M0 null modes (constant rate model) to detect different ω across lineages, and site models M2a (nearly neutral) and M8 (beta and ω) were tested against the M1a (nearly neutral) and M7 (beta) / M8a (beta and ω fixed to 1) respectively to detect variable selective pressures among sites. The likelihood ratio test (LRT) was used to evaluate the best evolutionary model. However, the PAML analysis does not consider recombination, so we also used the genetic algorithms for recombination detection (GARD), found in HyPhy, to check for recombination [48]. The recombination parameter *C* [49] was used to evaluate the degrees of recombination using DnaSP v.5 [43]. The fixed effects likelihood (FEL) and random effect likelihood (REL) models [50] in HyPhy were used to detect the signatures for the positive selection of specific codons, and the mixed-effects model of evolution (MEME) and branch-site REL (BSR) analysis were used to find the probable episodic selection events on subsets of lineages [51]. Alterations to site-specific amino acid properties through the evolutionary process were diagnosed using the methods followed by Conant et al. [52] and Atchley et al. [53], and the property-informed models of evolution (PRIME) model. Each individual null model (no property change) was compared to the full model by LRT using the Chi-square distribution to assess significance.

## Results

The *CHS* and *UFGT* partial sequences from *Scutellaria* obtained in this study had alignment lengths of 756 bps (252 codons) and 741 bps (247 codons), respectively. All sequences belonged to an exon. These two genes were found to be analogs of *TRANSPARENT TESTA 4* (*TT4*, E-value = 3 × 10^−137^) and *UDP-glucosyl transferase 73* (*UGT73*, E-value = 2 × 10^−11^), respectively, after a tBLASTx search using the *Arabidopsis thaliana* genome. The number of *CHS*/*UFGT* variable sites, parsimonious informative sites, and singletons were 243/227, 159/192, and 84/35, respectively.

**Fig. 1:**
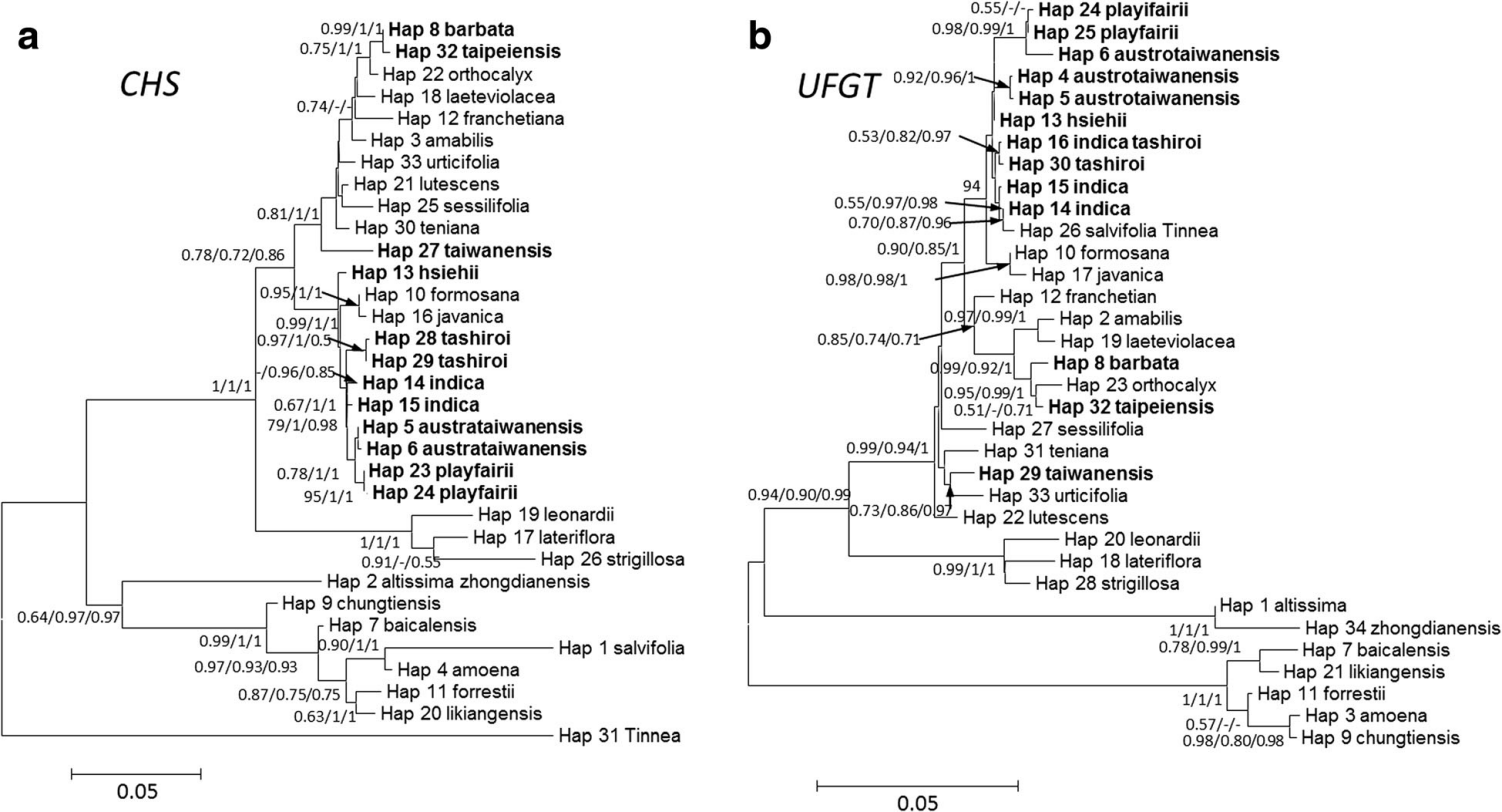
Neighbor-joining analyses of the **a** *CHS* gene and **b** the *UFGT* gene in selected *Scutellaria* species. These two trees have different topologies, which may be due to evolution under different selective pressures. Bold operational taxonomy units (OTUs) are native to Taiwan. Bootstrap value from Neighbor-joining and Maximum likelihood and posterior probability from Bayesian inference were labeled on the node

Phylogenetic tree reconstructed from different methods (neighbor joining, maximum likelihood, and Bayesian inferences) revealed similar topology in each single gene. However, gene trees of *CHS* and *UFGT* are different in phylogenetic topologies (Fig. 1). The *UFGT* sequence of the outgroup *Tinnea rhodesiana* was identical to a Turkish endemic species, *S. salviifolia,* and was grouped with *S. indica,* which is widespread in East, Southeast, and South Asia. Four recently divergent species: *S. indica*, *S. tashirroi*, *S. austrotaiwanensis*, and *S. playfairii,* were closely grouped within a clade in both *CHS* and *UFGT* gene trees, as was the recently identified species *S. hsiehii,* despite low bootstrapping supporting values (Fig. 1). The evolution of skullcaps corolla colours were then mapped in the phylogenetic trees for inferring ancestral states. Under the best fitted model (Bayarea-like, lnL = -45.675, weighted AIC = 0.999, Additional file 1: Table S2), the blue corolla colour was most likely to be ancestral state during divergence of skullcaps. Most of the corolla colour transitions were found to be in the terminal branch, including Taiwanense species (Additional file 1: Figure S1), suggesting that the adaptation plasticity of corolla colours might act on terminal lineages, including Taiwanese taxa, instead of ancestry.

The averaged ω values for *CHS* and *UFGT* were 0.0269 and 0.5656, respectively. We compared the ω values for *CHS* and *UFGT* in Taiwanese species to non-Taiwanese species to ascertain whether the differential selective force act on Taiwanese (island) *Scutellaria* or just reflected a normal trend in anthocyanin evolution. The relative rate of ω values were calculated using outgroup *Tinnea*. Relative ω values were significantly different between *CHS* and *UFGT* comparisons (bootstrap hypothesis test for equality, *P* < 2.2e-16). Furthermore, the relative ω values for both genes are significantly different between Taiwanese and non-Taiwanese *Scutellaria* (two-tailed paired-*t* test, *P* = 0.013 for *CHS* and < 2.2e-16 for *UFGT*, Additional file 1: Table S3). The relative ω values of *CHS* were significantly higher in non-Taiwanese *Scutellaria* (one-tailed paired-*t* test, *P* = 0.006) while the relative ω values of *UFGT* were significantly higher in Taiwanese *Scutellaria* (one-tailed paired-*t* test, *P* < 2.2e-16). This suggested that there was a higher proportion of diversifying selection or relaxation of selective constraints in *UFGT* of island species, while there was a pervasive purifying selection in *CHS* of island species.

**Fig. 2:**
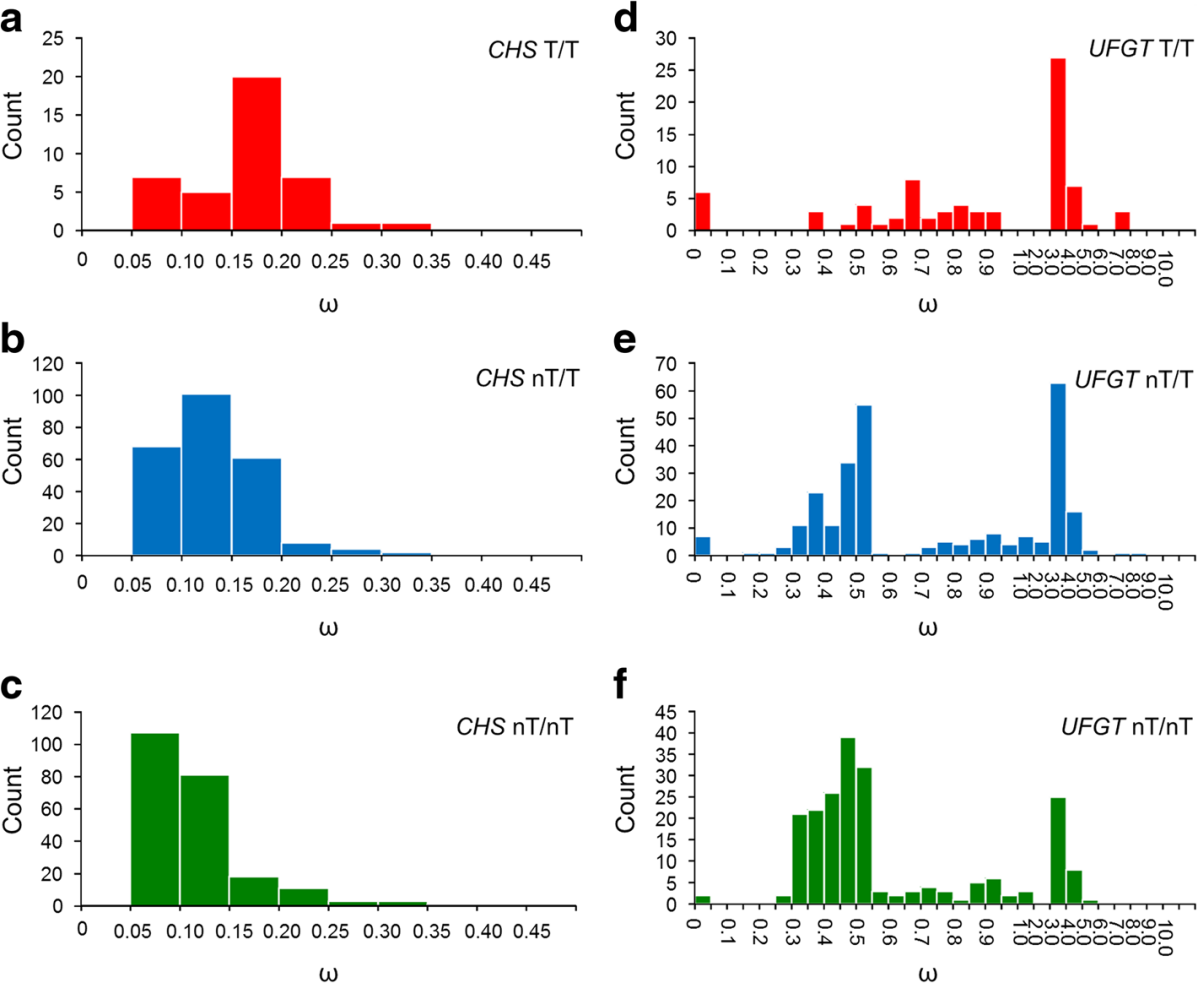
Distribution of ω values between Taiwanese lineages (T/T), between non-Taiwanese and Taiwanese lineages (nT/T), and between non-Taiwanese lineages (nT/nT) in *CHS* (**a**–**c**) and in *UFGT* (**d**–**f**)

**Fig. 3:**
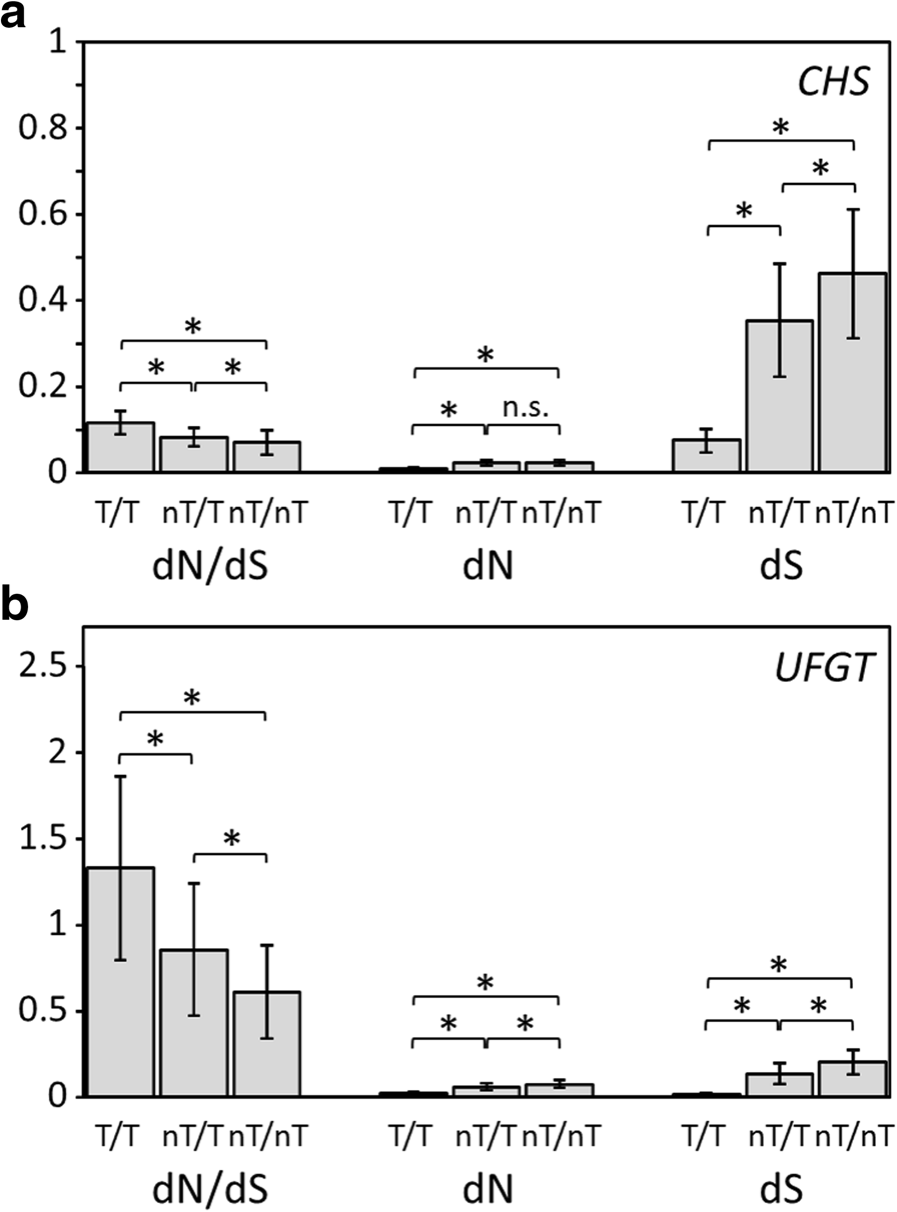
Comparisons of the average dN/dS ratios (ω), and dN and dS values for **a** *CHS* and **b** *UFGT* between Taiwanese lineages (T/T, first bar), non-Taiwanese and Taiwanese lineages (nT/T, second bar), and non-Taiwanese lineages (nT/nT, third bar). Capped lines indicate SE. All pair comparisons are significantly different (*P* < 0.001) except for the *CHS* dN value comparison between nT/T vs. nT/nT (*P* = 0.154)

The pairwise ω values for *CHS* between different Taiwanese lineages (T/T), between non-Taiwanese species and Taiwanese species (nT/T), and between lineages of non-Taiwanese species (nT/nT) were mostly within the range 0.15–0.20, 0.10–0.15, and 0.05–0.10, respectively (Fig. 2a–c). The distributional differences for ω were reflected in the statistical differences between the average ω values. The average ω for *CHS* was higher in T/T (ω = 0.117 ± 0.027) than in nT/T (ω = 0.083 ± 0.022, *P* = 0.0001) and nT/nT (ω = 0.07 ± 0.028, *P* = 1.986 × 10^−6^). A significantly higher ω for *UFGT* was also found in Taiwanese lineages (T/T: ω = 1.328 ± 0.533), nT/T (ω = 0.856 ± 0.384, *P* = 0.0004) and in nT/nT (ω = 0.612 ± 0.271, *P* = 2.815 × 10^−7^) (Fig. 3). This is because the *UFGT* ratio (ω < 1) in Taiwanese lineages (T/T) was far less than in the non-Taiwanese lineages (nT/T and nT/nT) (Fig. 2d–f). The relatively high ω values for the T/T lineages were also revealed in the ω vs. dS plot, which showed that the T/T lineages had a relatively small dS compared to the nT/T and nT/nT lineages (Additional file 1: Figure S2). Contrast results of slower evolutionary rate of *CHS* in relative rate test and higher pairwise ω for *CHS* in Taiwanese species is probably because small dS may elevate ω during pairwise comparison.

MK tests also reveal no fixed nonsynonymous substitution in *CHS* but slightly higher nonsynonymous fixed substitution in *UFGT* of Taiwanese sister species pairs, although variation is too limited to conduct statistical test (Additional file 1: Table S4). This suggested that *UFGT* of Taiwanese *Scutellaria* species suffered from higher divergent selective pressure from other species, but such divergent selection is none or less in *CHS* of Taiwanese *Scutellaria* species. Nonsignificant results of HKA test indicated no deviation from neutrality, suggesting no balancing or positive selection acting on both *CHS* and *UFGT* in either Taiwanese or non-Taiwanese *Scutellaria* species (Additional file 1: Table S5). However, such results could be biased due to homogeneous evolving in these two tested genes rather than rejection of positive selection [54].

Significant geographic structure can be detected in AMOVA in both *CHS* and *UFGT* (Additional file 1: Table S6, *P* < 0.0001 and 0.00684, respectfully), meaning that the Taiwan island plays an isolated environment for the independent evolution of both *CHS* and *UFGT*. In other words, the genetic diversification of *CHS* and *UFGT* in Taiwanese *Scutellaria* species is not or less affected by the gene flow from species of other places. Due to elimination of possibilities of ancestral polymorphisms and influence of immigrated genes, we hypothesized that the sources of high polymorphisms of ABP genes of Taiwanese species is driven by natural selection.

**Fig. 4:**
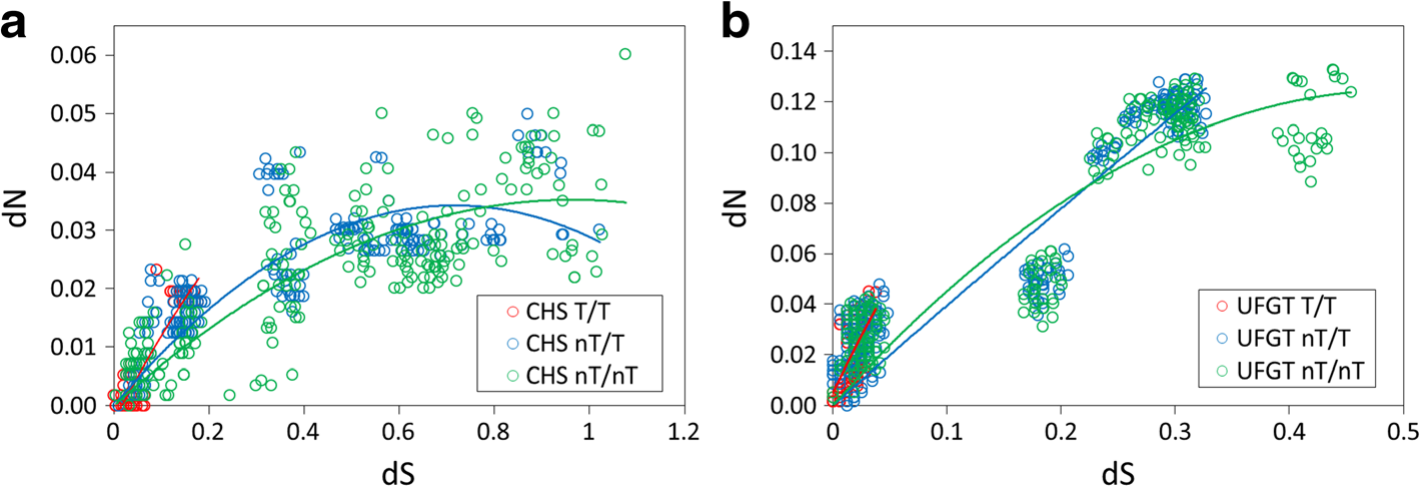
dN vs. dS plots of **a** *CHS* and **b** *UFGT*. Regression lines reveal the rate saturation of amino acid change in non-Taiwanese lineages

According to the comparison of dN and dS, we suggested higher proportion of diversifying selection pressure on *UFGT* lineages in island species and pervasive purifying selection or selectively constraints on *CHS* lineages. The smaller dS for both *CHS* and *UFGT* also reflected relatively recent diversification in these island lineages. The dN-vs.-dS plot revealed that regression line slopes were lower for the nT/T and nT/nT comparisons (Fig. 4), and that the nT/T and nT/nT lines for *CHS* (Fig. 4a) and the nT/nT line for *UFGT* (Fig. 4b) reached a plateau phase, which suggested that there was a saturation of mutations in amino-acid replacement.

**Fig. 5:**
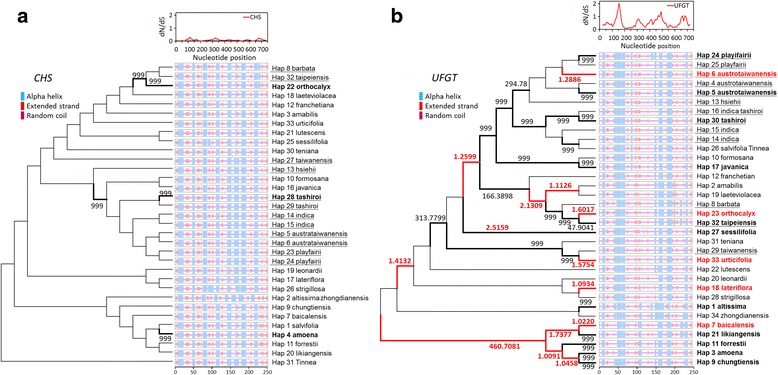
Results of the free-ratio model test and secondary structure predictions. **a** *CHS* and **b** *UFGT*. The branches with estimated ω > 1 are indicated in bold, and those with ω > 1 and dS > 1 are marked in red. The numbers beside the branches are the estimated ω values (only ω > 1 are shown). Haplotypes of Taiwanese species are underlined. Scale bars indicate the position of amino acid sequences

The selection may not act equally on all regions of sequences [55]. Therefore, selective constraints could have masked positive selection signals. We performed a sliding window analysis on the ω values between the lineages of Taiwanese species and non-Taiwanese species to examine whether selective pressure differentiates Taiwanese species from others. The results showed that *CHS* had evolved under purifying selection (ω < 1), but *UFGT* showed a dramatic rise in ω at the 145–165 bp and 450–500 bp nucleotide positions (Figs. 5a, b). We then predicted the secondary structures of *CHS* and *UFGT* and found that the secondary structure of *CHS* was mainly conserved*,* except for small fractions in certain species, e.g., Hap 2 (*S. altissima* and *S. zhongdianensis*) and Hap26 (*S. strigillosa*) (Fig. 5c). In contrast, *UFGT* had more diversified regions in its secondary structure (Fig. 5d). Overall, more lineages and gene regions with signature of positive selection (ω > 1) can be found in *UFGT* than *CHS*.

To further validate the inference of selective constraints on the evolution of *CHS* and of positive Darwinian selection on the diversity of *UFGT*, both branch and site models were tested using the codeml program. The LRT results for *CHS* revealed that the constant (M0) model was rejected by the free-ratio model (2ΔL = 123.048, df = 63, *P* = 2.39 × 10^−6^). Five branches of ω > 1 were detected and three of the five were at the terminal branches of *S. amoena* (haplotype 4), *S. orthocalyx* (haplotype 22), and *S. tashiroi* (haplotype 28) (Fig. 5). However, the ω values of these five positively selected branches were extremely large (ω = 999) as dS = 0, and these could be false positives. The site null models that limited all codons to ω ≤ 1 (M1a, M7, and M8a) cannot be rejected by the corresponding positive selection model (Table 1). In contrast to the few positively selected branches and the non-positively selected codons for *CHS*, *UFGT* had more branches with ω > 1 under the free-ratio model (2ΔL = 81.226, df = 65, *P* = 0.012) than under the M0 model, and the site-model tests showed that the M1a model was rejected by M2a (2ΔL = 20.361, df = 2, *P* = 1.90 × 10^−5^), and the M7 and M8a models were rejected by M8 (2ΔL = 23.390, df = 2, *P* = 4.17 × 10^−6^ and 2ΔL = 20.326, df = 1, *P* = 3.41 × 10^−6^, respectively). Three codons (47 N, 162 M, and 163R) were inferred to be *ω* > 1 with a posterior probability > 0.95 in M2a and > 0.99 in M8, by Bayes empirical Bayes analysis.

**Table 1:** Summary of codon-based model analyses of PAML for *CHS* and *UFGT* of *Scutellaria* species

Gene	Model	np	L	ω	Parameters	2ΔL	*P*	PS site (Pr ω > 1)
*CHS*	M0	66	−3401.398	0.0269	ω = 0.02694	123.048	2.39E-06	
	free ratio	129	−3339.873					
	M1a	67	−3377.238	0.0467	ω0 = 0.01830 (p0 = 0.97111) ω1 = 1.000 (p1 = 0.02889)	0.000	NA	
	M2a	69	−3377.238	0.0467	ω0 = 0.01830 (p0 = 0.97111) ω1 = 1.000 (p1 = 0.02889) ω2 = 35.71235 (p2 = 0)			
	M7	67	−3350.937	0.0301	α = 0.11018, β = 2.88094	0.062	0.485	
	M8a	68	−3350.906	0.0308	α = 0.11218, β = 3.09870 p0 = 0.99761 p1 = 0.00239; ω1 = 1.000	0.000	NA	
	M8	69	−3350.906	0.0308	α = 0.11218, β = 3.09870 p0 = 0.99761 p1 = 0.00239; ω1 = 1.000			
*UFGT*	M0	68	−3020.739	0.5656	ω = 0.56561	81.226	0.012	
	free ratio	133	−2980.126					
	M1a	69	−2961.023	0.4144	ω0 = 0.05827 (p0 = 0.62181) ω1 = 1.000 (p1 = 0.37819)	20.361	1.90E-05	
	M2a	71	−2950.843	0.6217	ω0 = 0.09111 (p0 = 0.63671) ω1 = 1.000 (p1 = 0.24187) ω2 = 2.65082 (p2 = 0.12142)			47 N (0.965) 162 M (0.963) 163R (0.996)
	M7	69	−2962.585	0.4105	α = 0.02259, β = 0.03044	23.390	4.17E-06	
	M8a	70	−2961.053	0.4144	α = 6.26619, β = 99 p0 = 0.62235 p1 = 0.37765; ω1 = 1.000	20.326	3.41E-06	
	M8	71	−2950.890	0.6198	α = 0.28974, β = 0.72287 p0 = 0.84684 p1 = 0.15316; ω1 = 2.46858			40 L (0.958) **47 N (0.993)** 111E (0.957) 112A (0.971) 114E (0.985) 131 L (0.964) 132S (0.975) **162 M (0.994)** **163R (0.999)** 171 K (0.957)

L, Log-likelihood of the data; ω, Mean dN⁄ dS ratio for the entire gene; NA, not available; the PS sites marked in boldface are the amino acids also being detected to be positively selected under the M2a model

The inference of positive selection by PAML is usually an argument for not considering the effect of recombination. Therefore, we estimated the recombination rate by parameter *C* [49] and used GARD to find the breakpoints in recombination events. The KH topological test was used to check the topological incongruence of the trees on both sides of the breakpoint fragments. In *CHS*, *C* is 6.7 per gene and 0.0089 per site, and one breakpoint at codon 381 (ΔAIC_c_ = 84.3502) was found to cause different topologies between segments (*P* < 0.05 in the KH test) by GARD. In *UFGT*, *C* was smaller than for *CHS* (*C* = 1.8 per gene and 0.0024 per site), and no evidence of recombination was found after using the KH test in the GARD analysis. These results indicated that the *CHS* gene experienced more recombination events than *UFGT*.

To reduce recombination effects on the inference of positive selection, the FEL and REL models were used to re-estimate the ω of the codons. In *CHS*, 136 and 113 negatively selected codons (ω < 1) were detected by FEL at the 0.05 significance level and by REL at Bayes Factor = 50, respectively, but neither model detected sites that had been subjected to pervasive positive selection (ω > 1). In *UFGT*, three sites (codons 131, 132, and 163) and 12 sites (codons 27, 40, 47, 111, 112, 114, 131, 132, 147, 162, 163, and 171) were shown to have evolved under pervasive positive selection by the FEL and REL models, respectively. Among these 12 positively selected sites detected under REL, only three sites had posterior probabilities > 0.99 (codons 47, 162, and 163). Thirteen negatively selected codons were detected in FEL, but no codon was negatively selected by REL.

In addition, we used the MEME model to test whether specific codons were positively selected on specific lineages, which is defined as episodic positive selection. One in *CHS* and six in *UFGT* codon sites were detected under episodic positive selection at the 0.05 significance level (in *CHS*: codon 39, *P* = 0.005; in *UFGT* codon 8, *P* = 0.035; codon 38, *P* = 0.011; codon 132, *P* = 0.048; codon 163, *P* = 0.015; codon 218, *P* = 0.0003; and codon 236, and *P* = 0.039), respectively (Additional file 1: Figure S3, S4). With the exception of codon 8, the other five codons in *UFGT* that showed episodic positive selection were all found in *S. zhongdianensis* (Additional file 1: Figure S4). The episodic positive selection pressure on *UFGT* was also detected in the lineage of *S. zhongdianensis* under the BSR model, which suggested that 1.68 % of codon sites were positively selected (ω = 155.10, *P* = 0.009 using the Holm-Bonferroni method). No branches of *CHS* were subject to episodic positive selection (*P* < 0.05) in the BSR analysis, suggesting that there is no positive selection acting on *CHS* of Taiwanese lineages. The PAML and HyPhy analyses suggested that the evolution of *CHS* is probably under selective constraint and has slow amino acid replacement rates, while multiple episodes of positive Darwinian selection seem to drive *UFGT* diversity.

We also used PRIME to look for adaptive changes to amino acid properties by the evolutionary process using Conant et al.’s [52] and Atchley et al.’s [53] methods (Table 2). Property alterations in side-chain volume were detected at the 39^th^ (*P* = 0.010) and 146^th^ codons (*P* = 0.016) in *CHS*, and there was a polarity change at the 142^nd^ codon (*P* = 0.040). The 104^th^, 120^th^, and 146^th^ codons showed conserved hydropathy (*P* = 0.041), chemical composition (*P* = 0.026), and refractivity/heat capacity (*P* = 0.049) properties, in *UFGT*; while adaptive changes in the charge/iso-electric point was detected at the 31^st^ codon (*P* = 0.024) and a conserved isoelectric point at the 192^nd^ codon (*P* = 0.044).

**Table 2:** Results of Property Informed Models of Evolution (PRIME) showed site-specific amino acid properties in adaptive modification (positive values) and conservation (negative values)

		Conant et al.’s method [36]				
Gene	Codon	Chemical composition	Polarity	Volume	Isoelectric point	Hydropathy
*CHS*	39	4.697 (0.193)	−3.362 (1)	**−13.973 (0.010)**	15.523 (0.102)	12.408 (0.134)
*CHS*	104	1.561 (1)	**−20 (0.040)**	−1.285 (0.770)	20 (0.616)	**16.325 (0.041)**
*CHS*	120	**20 (0.026)**	16.656 (0.543)	−8.149 (1)	1.838 (1)	−2.734 (1)
*UFGT*	192	0.278 (1)	−5.116 (0.094)	0.819 (1)	**17.008 (0.044)**	0.724 (1)
		Atchley et al.’s method [37]				
Gene	Codon	Polarity index	Secondary structure factor	Volume	Refractivity/Heat Capacity	Charge/Isoelectric point
*CHS*	146	3.337 (0.643)	11.474 (0.343)	**−4.393 (0.016)**	**8.551 (0.049)**	0.814 (0.646)
*UFGT*	31	13.932 (1)	0.635 (0.904)	2.079 (0.194)	−0.531 (1)	**−4.879 (0.024)**

The values in the parentheses are the corrected *P*-value for the likelihood-ratio test that the property values ≠ 0. Significant values are indicated in bold

## Discussion

Biologists have debated on adaptive evolutionary morphological change are more likely to occur in structural enzyme or regulator [17, 56]. Signatures of positive selection on *R2R3-MYB* genes that function on regulating ABP genes have been found in *Scutellaria* [10]. In this study, *CHS* and *UFGT* are genes that are upstream and downstream of the ABP, respectively. They are structural enzymes and responsible for the biosynthesis of anthocyanin. Our results supported the previous suggestions of heterogeneous evolutionary rates in these downstream and upstream genes [2–4, 57]. However, most previous studies suggested that pervasive purifying selection pressures on these genes restricted anthocyanin diversity, except for certain episodic positive selections or relaxed constraints on *CHS* or *UFGT* genes [3]. In our analyses, genetic signatures for positive selection were detected in *UFGT* by the site model and free-ratio model produced by PAML, the FEL and REL models produced by HyPhy, the pairwise dN/dS analysis, and the sliding window analysis. Five *UFGT* codons, 47 N, 131 L, 132S, 162 M, and 163R, were detected in two or all of site-specific models and in the sliding-window analysis of ω (Fig. 5b). In particular, 131 L, 132S, 163R were also detected in FEL constructed by HyPhy, which was suggested to be less false-positively than the other two models [50]. These codons mostly corresponded to the absence or presence of an α-helix in the secondary structure (Fig. 5d). The regions display a structure with β-sheet interspersed among α-helixes that are known as Rossmann-like folds, and form a cleft for substrate binding [58]. The absence or presence of an α-helix may alter the fold structure and may further affect substrate specificity [58]. However, PRIME inferred that while these positively selected *UFGT* codons did not have any estimated property changes, the 31^st^ codon had an altered charge/isoelectric point (Table 2). This suggested that it is selective pressure drives protein conformation divergence in *UFGT,* rather than amino acid properties.

The inference that positive selection drives divergence in UFGT conformation, rather than functional divergence, in *Scutellaria* species is plausible because the anthocyanins are a group of metabolic products involved in stress tolerance, UV resistance, and pollination [14–16, 59]. Such ecologically important products are usually functionally conserved (translational robustness), but show changes in functional (translational) efficiency [60, 61]. A conformation change in a gene sometimes contributes to the adaptive function of meiotic recombination [62, 63]. However, the small recombination rate of *UFGT* (*C* = 0.0024 per site and no evidence of recombination by the KH test) excludes the probability of adaptive allele replacement through gene conversion. These small or no recombination and gene conversion events and results of HKA test also reject the hypothesis of balancing selection, which usually retains ancestral polymorphism by recombination and is also present in long-lasting trans-species polymorphism [64–66]. Therefore, we suggest that the multiple amino acid replacements with higher ω values among *UFGT* lineages (Fig. 5d, Additional file 1: Figure S5) are the relicts of selection for advantageous mutations accumulated throughout evolutionary history.

The average pairwise ω values for at least *UFGT* in the Taiwanese species were larger than those for non-Taiwanese species, and their dN and dS values are significantly smaller than those of the non-Taiwanese species (Additional file 1: Figure S2 and Table S3), which suggests recent divergent selection. The recent divergence is consistent with the inference of recent positive selection of skullcaps according to the phenomenon of corolla colour transitions on terminal or Taiwanese branches (Additional file 1: Figure S1). Our previous study has shown recently paleoclimate-related species divergences of Taiwanese skullcaps within 0.2 Mya [19]. Here, we provided the other evidence regarding the recent natural selection on genes of ecological traits on these island skullcaps. However, the dN between Taiwanese species is not as small as the dS, especially for *UFGT*, when compared with long-term divergent species (i.e., non-Taiwanese species) (Additional file 1: Figure S2). This indicates rapid accumulation of non-synonymous mutations in Taiwanese species, and also implies that non-synonymous mutations are not be continually accumulated, which would reduce the high mutational load [67, 68]. In contrast, synonymous mutations are easily accumulated when mutational load was low [69]. If this suggestion is true, we could expect a decrease in the rate of non-synonymous mutation compared to the synonymous mutation rate between species with longer divergent times (e.g., between non-Taiwanese species or between Taiwanese and non-Taiwanese species), which would reduce the damage caused by mutational load. To test this hypothesis, we drew dN-vs.-dS plots. The results showed that the non-Taiwanese lineages (nT/T and nT/nT) had shallower slopes than the Taiwanese lineages (T/T) for both *CHS* and *UFGT*. Some even reached a plateau phase (i.e., rate saturation of amino acid change) (Fig. 4). In contrast, the recently diverged Taiwanese species had relatively steep slopes for both *CHS* and *UFGT*, which suggested that there was a rapid accumulation of non-synonymous mutations at the beginning of species divergence. The results for the recent selection of varied amino acids by recently diverged species supports the hypothesis of late-burst diversification in Taiwanese *Scutellaria* species [19] and local diversifying selection in Taiwanese species.

Geographic structuring variation has been illustrated in both genes, mild proportion of variation can be explained by geographic structure (between Taiwanese and non-Taiwanese, 20.73 % and 17.4 % in *CHS* and *UFGT* respectfully, Additional file 1: Table S6). The minor but significantly structure of variation in downstream ABP genes has also been reported in *Ipomoea* [4]. Besides, the higher dN/dS ratio for ABP genes in Taiwanese species (Fig. 3, especially *UFGT* in Additional file 1: Table S3) implies that local selection is diversifying Multiple-time origins for Taiwanese species [19] is an alternative explanation for the high divergence in functional genes. The ω distribution (Fig. 3) was compared to ascertain which of these two hypotheses (i.e., local diversifying selection vs. multiple-time originations) was more likely. If the selection hypothesis were true, a skew towards higher ω values in Taiwanese species rather than non-Taiwanese species would be expected. Alternatively, if the multiple-origin hypothesis is true, we would expect the ω distribution to be similar between Taiwanese and non-Taiwanese species (i.e., functional gene divergence was driven by similar selective pressures). There is a slight skew towards higher ω values in *CHS* and an obvious low frequency of small ω values in the Taiwanese species (Fig. 2), which supported local diversifying selection.

Taken together, the Taiwanese species had higher amino-acid replacement rates in *UFGT* (Additional file 1: Table S3), which suggested that rapid divergence counteracted the selective sweeps of different selective pressure in ABP genes amongst these phylogenetically close species. Species within islands have higher encounter rates than continental species or species between islands, which increases the competitive pressure on species expansion (cf. [70]). Physiological divergence accelerates niche divergence and reduces the cost of species competition [71]. This explains the rapid divergence of ABP genes in Taiwanese species compared to other species (Fig. 3). Both the *CHS* and *UFGT* genes sequenced in this study were expressed in the inflorescence buds, according to the tissue-specific RNA-seq of *S. indica*, *S. tashiroi*, *S. playfairii*, and *S. taiwanensis* (unpublished data), which indicated that these two genes are involved in flower-color diversity [16]. The *UFGT* expression level is approximately 0.8 ~ 1.3 % that of *CHS* (unpublished data). It has been suggested that this highly expressed gene evolved slowly [72–74] and that there is a positive correlation between dN and expression specificity [72, 74]. Hence we speculate that the positive selection pressure on *UFGT* and the rapid divergence in Taiwanese *Scutellaria* species are probably related to petal color divergence, which also affects pollination [15, 16, 75]. In addition to *UFGT*, positive selection signals were also detected in the R2R3 type *MYB11* and *MYB16* genes in Taiwanese *Scutellaria* species [10], which help regulate the development of inflorescence axillary meristems [76, 77], and are the microRNA involved in filament development and pollen maturation [78], respectively. This study, together with previous studies, indicates that genes involving in floral or inflorescence types and colors may be key adaptive characters and may be associated with the rapid diversification of plant species.

## Conclusions

The evolution of ABP genes is an important issue because of its ecological relevance to stress resistance and pollination [14–16, 59]. Several studies have indicated genetic conservation in genes that are upstream of ABP genes, whereas there has been a relaxation of selective constraints in downstream genes [2–4]. The results of this study further suggested that *UFGT* was positively selected in *Scutellaria*, especially in those phylogenetically closed island species in Taiwan. Such gene divergence was due to changes in protein conformation rather than variations in DNA-binding domains, which prevents mutational load when there is a rapid accumulation of non-synonymous mutations. A previous investigation suggested that there was positive selection of the R2R3-MYB transcription factors, which regulate the transcription of ABP genes. This study supplements the translational information on positive selection signatures for ABP genes. Although less direct evidence supports the hypothesis that there are positive selection pressures on the Taiwanese species, significantly higher ω values imply that rapid divergence of the ABP genes reduced the competitive pressures caused by positive selection within island species. Furthermore, our study extended previous reports with conclusion of relaxation of selective constraints in downstream ABP genes [2, 4], and illustrated that geographic heterogeneity may drive different evolutionary scenarios between different order of the same pathway genes. In this context, our study strongly suggested that floral diversity is an integrative mechanism combining both intrinsic (genetic) and extrinsic (ecological) factors.

## Additional file

Additional file 1: Table S1. List of *Scutellaria* species used in this study. **Table S2.** Likelihood statistics results of ancestral area reconstruction models implemented in BioGeoBEARS. **Table S3.** Paired *t* test of ω ratios calculated from relative rate test implemented in HyPhy. **Table S4.** McDonald and Kreitman test of *CHS* and *UFGT* among Taiwanese skullcap sister species pairs. **Table S5.** HKA test results of CHS and UFGT between Taiwanense and non-Taiwanese skullcap species. **Table S6.** AMOVA analysis of *CHS* and *UFGT* between Taiwanese and non-Taiwanese species. **Figure S1.** Mapping of the flower colours on a skullcaps phylogeny. Probability of ancestral state was mapped on the node. Colour in boxes corresponded to different flower colours. Blue: blue colours; Red: red colours; Yellow: yellow colours; Grey: white colours. **Figure S2.** The dN/dS (ω) vs. dS plots show a comparison of the ω distribution and the relative divergent times between Taiwanese species (T/T), non-Taiwanese and Taiwanese species (nT/T), and between non-Taiwanese species (nT/nT) for *CHS* (A–C) and *UFGT* (D–F). Horizontal lines in D–F indicate the boundary for ω = 1. **Figure S3.** Result of mixed effects model of evolution (MEME) analysis for the *naringenin-chalcone synthase* (*CHS*) gene. **Figure S4.** Result of mixed effects model of evolution (MEME) analysis for the *UDP-glucose:flavonol 3-O-D-glucosyltransferase* (*UFGT*) gene. (DOCX 1612 kb)
