# Supplementary material for: Diversifying selection of the anthocyanin biosynthetic downstream gene UFGT accelerates floral diversity of island Scutellaria species
Source: BMC Evol Biol. 2016 Sep 17;16:191. doi: 10.1186/s12862-016-0759-0 (PMC5027097; doi:10.1186/s12862-016-0759-0)
Supplement: Additional file 1: Table S1. — List of Scutellaria species used in this study. Table S2. Likelihood statistics results of ancestral area reconstruction models implemented in BioGeoBEARS. Table S3. Paired t test of ω ratios calculated from relative rate test implemented in HyPhy. Table S4. McDonald and Kreitman test of CHS and UFGT among Taiwanese skullcap sister species pairs. Table S5. HKA test results of CHS and UFGT between Taiwanense and non-Taiwanese skullcap species. Table S6. AMOVA analysis of CHS and UFGT between Taiwanese and non-Taiwanese species. Figure S1. Mapping of the flower colours on a skullcaps phylogeny. Probability of ancestral state was mapped on the node. Colour in boxes corresponded to different flower colours. Blue: blue colours; Red: red colours; Yellow: yellow colours; Grey: white colours. Figure S2. The dN/dS (ω) vs. dS plots show a comparison of the ω distribution and the relative divergent times between Taiwanese species (T/T), non-Taiwanese and Taiwanese species (nT/T), and between non-Taiwanese species (nT/nT) for CHS (A–C) and UFGT (D–F). Horizontal lines in D–F indicate the boundary for ω = 1. Figure S3. Result of mixed effects model of evolution (MEME) analysis for the naringenin-chalcone synthase (CHS) gene. Figure S4. Result of mixed effects model of evolution (MEME) analysis for the UDP-glucose:flavonol 3-O-D-glucosyltransferase (UFGT) gene. (DOCX 1612 kb) [file 12862_2016_759_MOESM1_ESM.docx]

**Table S1** List of *Scutellaria* species used in this study.

| Species | Distribution | Sample source |
| --- | --- | --- |
| *S. austrotaiwanensis* | Taiwan | Hengchun peninsula, Lilungshan, and Nanhua, Taiwan |
| *S. indica* | Taiwan | Awanda and Wulai, Taiwan |
| *S. playfairii* | Taiwan | Dahan trail, Wutai, and Wulu, Taiwan |
| *S. tashiroi* | Taiwan | Lanyu Island, Mugumuyu, Taroko, and Wulu, Taiwan |
| *S. taiwanensis* | Taiwan | Jin-Shui Camp, Taiwan |
| *S. barbata* | Taiwan | Yonghe Dist., Taipei, Taiwan |
| *S. taipeiensis* | Taiwan | Maokong, Taiwan |
| *S. hsiehii* | Taiwan | Renlun Logging trail,Taiwan |
| *S. amoena* | Southwestern China | Kunming Institute of Botany, China |
| *S. orthocalyx* | Southwestern China | Kunming Institute of Botany, China* |
| *S. laeteviolaceae* | Japan, China | Kunming Institute of Botany, China* |
| *S. franchetiana* | Southwestern China | Kunming Institute of Botany, China* |
| *S. amabilis* | Japan | Osaka Mt. Ikoma, Japan |
| *S. urticifolia* | Yunnan, China | Kunming Institute of Botany, China* |
| *S. lutescens* | Yunnan, China | Kunming Institute of Botany, China* |
| *S. sessilifolia* | Sichuan, China | Sichuan, China |
| *S. teniana* | Yunnan, China | Kunming Institute of Botany, China* |
| *S. formosana* | Southwestern China | Kunming Institute of Botany, China* |
| *S. javanica* | Southeast Asia | Chongshe Flower Market, Taiwan |
| *S. leonardii* | North American | Kunming Institute of Botany, China* |
| *S. lateriflora* | North American | B & T World Seed |
| *S. strigillosa* | Japan, Korea, China | Kunming Institute of Botany, China* |
| *S. chungtiensis* | China | Kunming Institute of Botany, China* |
| *S. alpina* | Europe alpine region | Chiltern Seed |
| *S. forrestii* | Southwestern China | Kunming Institute of Botany, China* |
| *S. baicalensis* | Northeastern Asia | B & T World Seed |
| *S. salvifolia* | Western Asia | Chiltern Seed |
| *S. altissima* | Europe | B & T World Seed |
| *S. likiangensis* | Yunnan, China | Kunming Institute of Botany, China* |
| *S. zhongdianensis* | Europe | B & T World Seed |
| *Tinnea rhodesiana* | South American | B & T World Seed |

*: provided by Dr. Chunlei Xiang, Kunming Institute of Botany.

**Table S2** Likelihood statistic results of ancestral area reconstruction models implemented in BioGeoBEARS. The best model is BayArea-like model.

|  | lnL | No. parameters | *d* | *e* | *j* | weighted AIC |
| --- | --- | --- | --- | --- | --- | --- |
| DEC | -63.568 | 2 | 0.0370 | 3.17E-03 | 0.000 | 4.61E-08 |
| DEC+J | -62.679 | 3 | 0.0328 | 1.00E-12 | 0.014 | 4.12E-08 |
| DIVALIKE | -63.986 | 3 | 0.0384 | 1.00E-12 | 0.001 | 1.12E-08 |
| DIVALIKE+J | -63.986 | 3 | 0.0385 | 1.00E-12 | 0.001 | 1.12E-08 |
| **BAYAREALIKE** | **-45.675** | **3** | **0.0151** | **1.00E-12** | **0.028** | **0.999** |
| BAYAREALIKE+J | -63.986 | 3 | 0.0385 | 1.00E-12 | 0.001 | 1.12E-08 |

*d*: dispersal rate

*e*: extinction rate
*j*: founder speciation rate

**Table S3** Paired *t* test of ω ratios calculated from relative rate test implemented in HyPhy. Two-tailed paired *t* test were conducted to test the equality between Taiwanese or non-Taiwanese skullcap species, and one-tailed paired *t* test were used to determine whether the evolutionary rate (ω) is greater in Taiwanese or non-Taiwanese skullcap species. The results revealed unequality of evolutionary rate in both genes, and that evolutionary rate of *CHS* is significantly greater in non-Taiwanese species while *UFGT* is significantly greater in Taiwanese species

|  | *P*_(HA:ωnt ≠ωt)_ | *P*_(HA:ωnt <ωt)_ | *P*_(HA:ωnt >ωt)_ |
| --- | --- | --- | --- |
| *CHS* | **<2.2e-16** | 1 | **<2.2e-16** |
| *UFGT* | **0.01255** | **0.00628** | 0.9937 |

H_A_: alternative hypothese for paired *t* test

ωnt: ω ratios calculated from non-Taiwanense skullcap species

ωt: ω ratios calculated from Taiwanense skullcap species

**Table S4** McDonald and Kreitman test of *CHS* and *UFGT* among Taiwanese skullcap sister species pairs. The two-way (fixed / polymorphic and synonymous / nonsynonymous substitutions) contingency table was listed below and the Fisher’s exact test was used to test for independence of the two factors: the synonymicity (synonymous or nonsynonymous) and the fixity (polymorphic or fixed). If the corresponding genes were selectively neutral, there would be no difference in proportion of synonymous and nonsynonymous substitution number between polymorphic and fixed categories, or too limited variation for analysis.

| ingroup - outgroup | fixed | | | polymorphic | | Fisher's exact test *P* value |
| --- | --- | --- | --- | --- | --- | --- |
| *CHS* | |  |  | |  | |
| *S. austrotaiwanensis – S. playfairii* | |  |  | |  | |
| Synonymous | | 3 | 2 | | nc | |
| Nonsynonymous | | 0 | 0 | |  | |
| *S. playfairii – S. austrotaiwanensis* | |  |  | |  | |
| Synonymous | | 3 | 2 | | nc | |
| Nonsynonymous | | 0 | 0 | |  | |
| *S. tashiroi – S. austrotaiwanensis* | |  |  | |  | |
| Synonymous | | 8 | 1 | | 0.2 | |
| Nonsynonymous | | 0 | 1 | |  | |
| *S. tashiroi – S. playfairii* | |  |  | |  | |
| Synonymous | | 11 | 1 | | 0.15 | |
| Nonsynonymous | | 0 | 1 | |  | |
| *S. barbata – S. taipeiensis* | |  |  | |  | |
| Synonymous | | - | - | | monomorphic | |
| Nonsynonymous | | - | - | |  | |
| *UFGT* | |  |  | |  | |
| *S. austrotaiwanensis – S. playfairii* | |  |  | |  | |
| Synonymous | | 0 | 4 | | nc | |
| Nonsynonymous | | 0 | 11 | |  | |
| *S. tashiroi – S. austrotaiwanensis* | |  |  | |  | |
| Synonymous | | 0 | 4 | | 1 | |
| Nonsynonymous | | 2 | 10 | |  | |
| *S. tashiroi – S. playfairii* | |  |  | |  | |
| Synonymous | | 3 | 0 | | 0.53 | |
| Nonsynonymous | | 5 | 2 | |  | |
| *S. barbata –S. taipeiensis* | |  |  | |  | |
| Synonymous | | 1 | 0 | | nc | |
| Nonsynonymous | | 3 | 0 | |  | |
| *S. taipeiensis-S. barbata* | |  |  | |  | |
| Synonymous | | 1 | 0 | | nc | |
| Nonsynonymous | | 3 | 0 | |  | |

nc: not calculable

**Table S5** HKA test results of *CHS* and *UFGT* between Taiwanense and non-Taiwanese skullcap species. The number of differences between (Divergence) or within (polymorphism) Taiwanese and non-Taiwanese species of each gene was listed below. The Chi-square test were conducted to test if the polymorphism with Taiwanese and non-Taiwanese species, and the divergence between them were all independent. Non-significant results (*P*>0.05) indicated selective neutrality or homogeneous evolutionary scenarios in the corresponding genes.

|  | *CHS* | *UFGT* |
| --- | --- | --- |
| Divergence of Taiwanese / non-Taiwanese | 59.08 | 50.37 |
| Polymorphism data of Taiwanese species | 60 | 48 |
| Chi-square = 0.007 *P*= 0.93 |  |  |
|  | | |
| Divergence of Taiwanese / non-Taiwanese | 59.08 | 50.37 |
| Polymorphism data of non-Taiwanese species | 208 | 212 |
| Chi-square = 0.014 *P*=0.91 |  |  |

**Table S6** AMOVA analysis of *CHS* and *UFGT* between Taiwanese and non-Taiwanese species. The genetic variations were compare among or within groups (Taiwanese and non-Taiwanese species). One thousand permutations were conducted to test whether variations were structured between groups (*F_ST_*). *P* value lower than 0.05 indicated significant genetic structure between Taiwanese and non Taiwanese species.

| source of variation | DF | sum of squares | variance components | percentage |
| --- | --- | --- | --- | --- |
| *CHS* |  |  |  |  |
| between Taiwanese and non-Taiwanese | 1 | 123.3 | 6.6 | 20.7 |
| within group | 30 | 751.4 | 25.0 | 79.3 |
| total | 31 | 874.8 | 31.6 |  |
| *F_ST_* =0.21 |  |  | *P*<0.001 |  |
| *UFGT* |  |  |  |  |
| between Taiwanese and non-Taiwanese | 1 | 105.1 | 5.0 | 17.4 |
| within group | 32 | 767.5 | 24.0 | 82.6 |
| total | 33 | 872.7 | 29.0 |  |
| *F_ST_* =0.17 |  |  | *P*=0.007 |  |

DF: degree of freedom


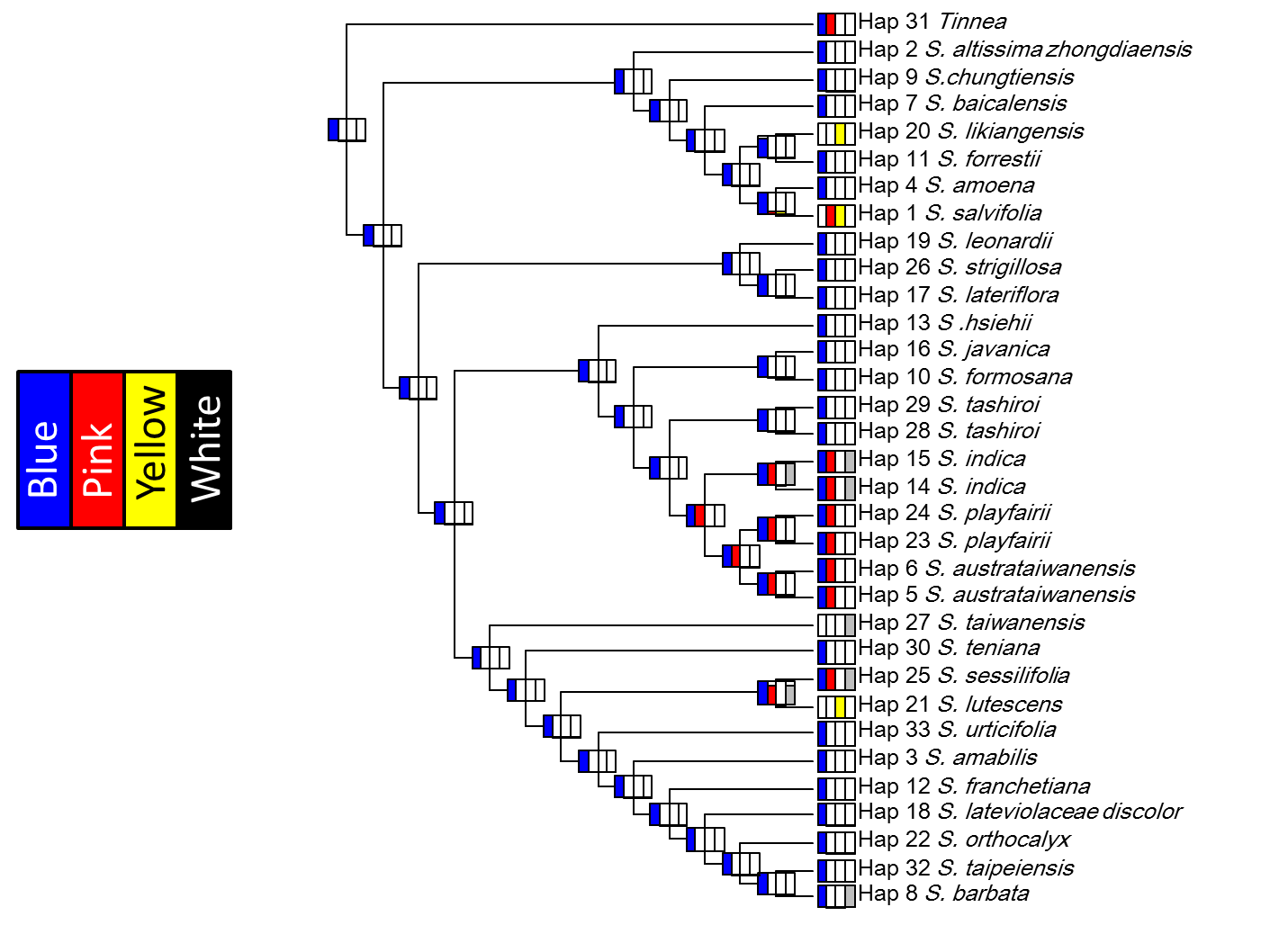


**Figure S1.** Mapping of the flower colours on a skullcaps phylogeny. Plot of per-character-state probabilities was provided on the node. It should be noted that most of the transition or gain of the character states were located in the terminal branches. Colour in boxes corresponded to different flower colours. Blue: blue colours; Red: red colours; Yellow: yellow colours; Grey: white colours.


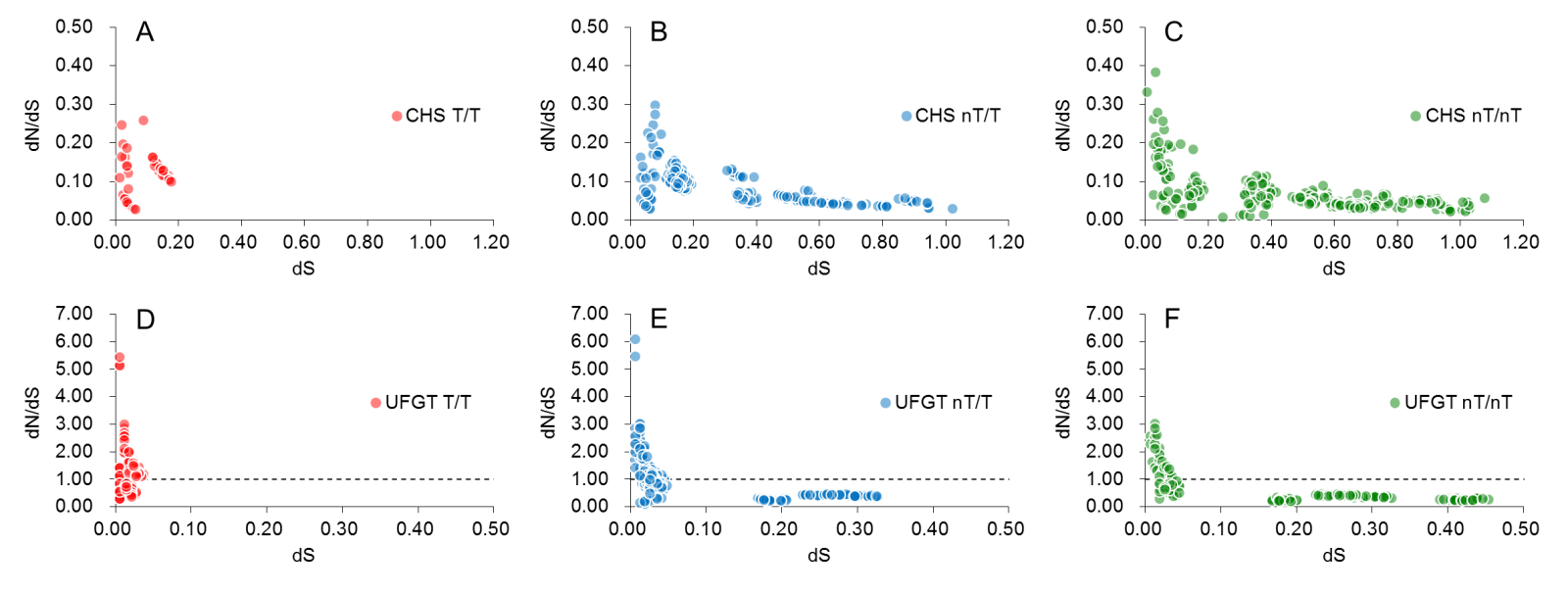


**Figure S2.** The dN/dS (ω) vs. dS plots show a comparison of the ω distribution and the relative divergent times between Taiwanese species (T/T), non-Taiwanese and Taiwanese species (nT/T), and between non-Taiwanese species (nT/nT) for *CHS* (A–C) and *UFGT* (D–F). Horizontal lines in D–F indicate the boundary for ω = 1.


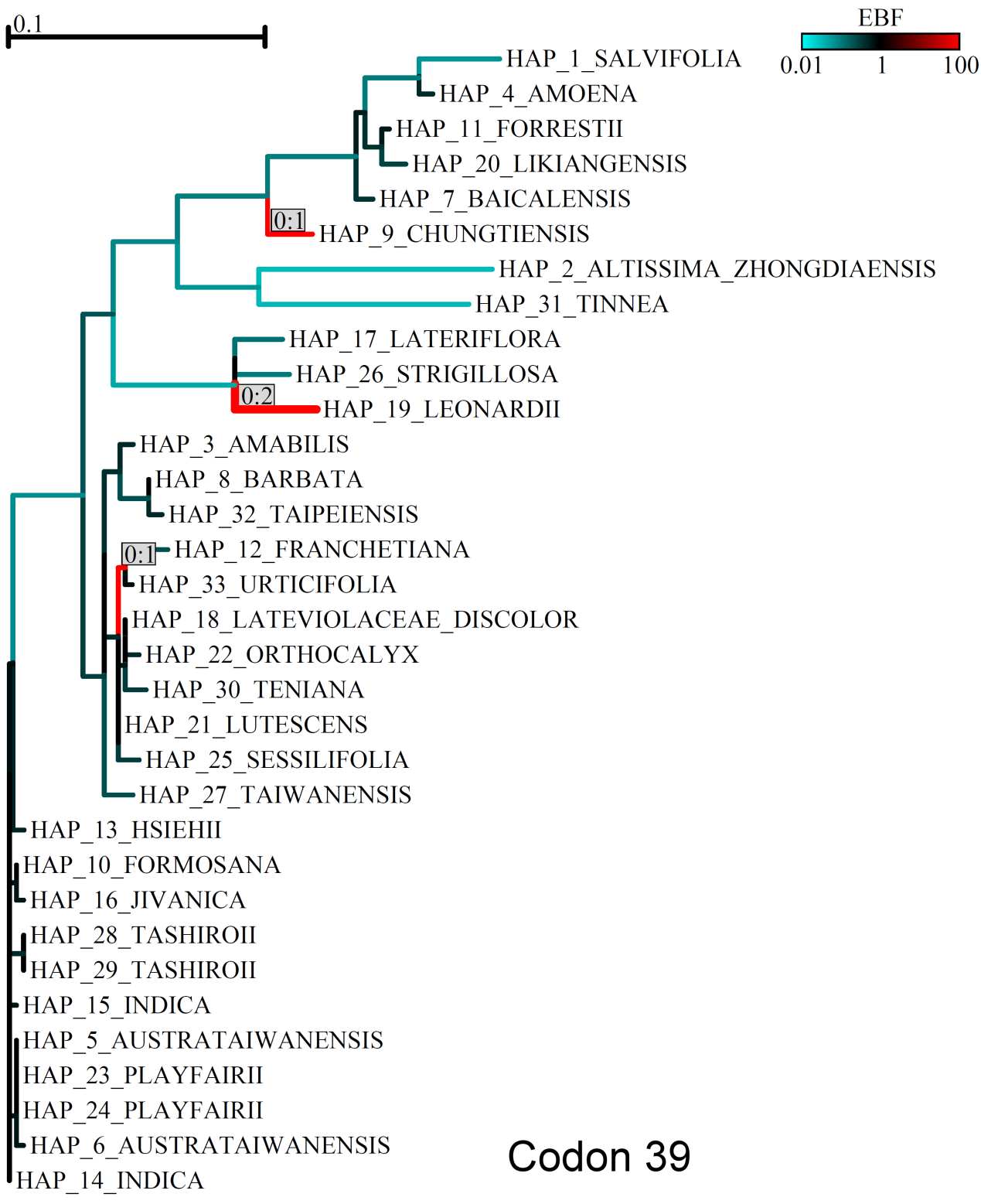


**Fig. S3** Result of mixed effects model of evolution (MEME) analysis for the *naringenin-chalcone synthase* (*CHS*) gene.


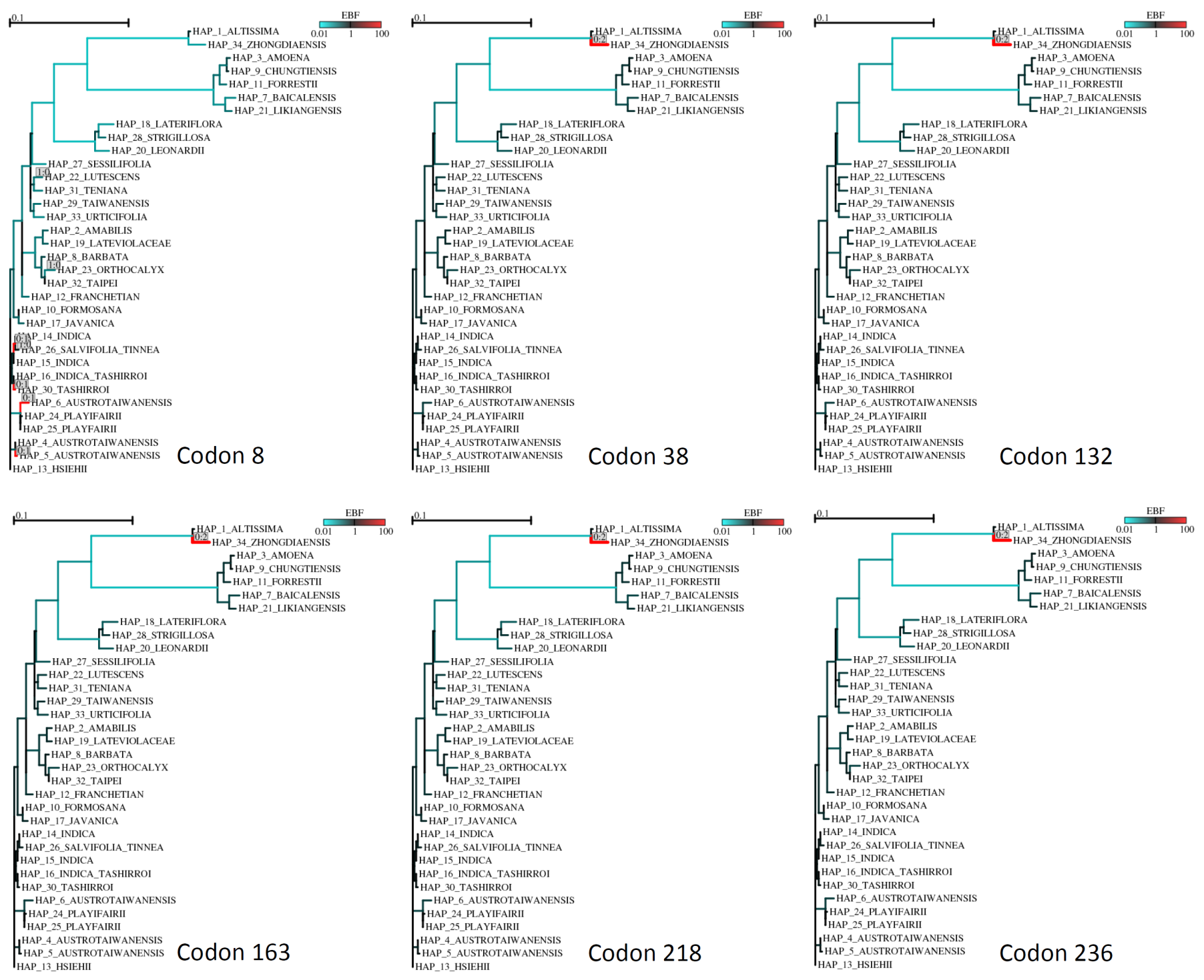


**Fig. S4** Result of mixed effects model of evolution (MEME) analysis for the *UDP-glucose:flavonol 3-O-D-glucosyltransferase* (*UFGT*) gene.
